# Patients with New-Onset Tumor of Severe Coronary Artery Disease May be at a Higher Risk of Arrhythmia

**DOI:** 10.1155/2021/1948624

**Published:** 2021-07-22

**Authors:** Palisha Alimu, Dezhi Yang, Huatao Zhou, Yan Wu, Huiping Guo

**Affiliations:** ^1^Department of Emergency Medicine, Sun Yat-sen Memorial Hospital, Sun Yat-set University, Guangzhou, China; ^2^Department of Cardiovascular Medicine, The People's Hospital of HeChi, Hechi, China; ^3^Department of Cardiovascular Medicine, Sun Yat-sen Memorial Hospital, Sun Yat-set University, Guangzhou, China; ^4^Department of Oncology Medicine, The People's Hospital of HeChi, Hechi, China

## Abstract

**Background:**

Arrhythmia is one of the causes of death in severe coronary artery disease patients who also suffered from cancer. Our research aims to compare the incidence of arrhythmia between severe coronary artery disease patient with and without new-onset tumor. *Methodology*. We enrolled 79 patients (December 2019–December 2020) with severe coronary artery disease in this study, and 40 of them were complicated with new-onset tumor. The details of all subjects were thoroughly obtained; the laboratory tests were implemented including creatinine before coronary angiography. The appraisal of the severity of coronary artery disease was applied by Gensini score. The cardiac inspection includes UCG, 12-lead ECG, and Holter monitor.

**Results:**

Among them, there were 40 patients in the experimental group and 39 patients in the control group. The difference at the baseline between the two sets of figures was not statistically significant (*P* > 0.05). The incidence of arrhythmia between the two groups was statistically significant (*P* < 0.05).

**Conclusions:**

The incidence of arrhythmia in severe coronary artery disease patients who were complicated with new-onset tumor was higher than that in patients with severe coronary artery disease alone, and attention should be paid to arrhythmia before tumor treatment.

## 1. Introduction

Although mortality rates of coronary heart disease and tumors have declined, they remain the leading causes of death in humans [1, 2]. Arrhythmia is very common in patients with coronary heart disease complicated with tumor [3]. Cognition and management of arrhythmia require an understanding of common etiologies and incidence [4]. Mechanisms of arrhythmia in cancer patients include direct cardiac involvement by cancer or cancer treatment-induced arrhythmia including radiation, chemotherapy, and surgery [5]. Coronary artery disease is also one of the risk factors for arrhythmia, and previous studies have shown that the heavier coronary lesions had higher incidence of arrhythmia and, at the same time, the worse prognosis [6]. Gensini scores are often used in clinical trials to assess coronary artery severity [7]. Few studies had been conducted on arrhythmia in cancer patients complicated by severe coronary artery disease. Improper recognition or treatment of arrhythmia may lead to adverse clinical consequences. Clinically, little attention has been paid to arrhythmia before tumor treatment. The study was aimed at comparing the difference of the two groups in the incidence of arrhythmia.

## 2. Patients and Methods

We conducted a controlled cross-sectional retrospective analysis. A total of 40 patients with new-onset tumor diagnosed in our hospital and coronary angiography suggesting severe coronary artery disease at the same time in hospital from December 2019 to December 2020 were selected, and 39 nontumor patients diagnosed with severe coronary artery disease undergoing coronary angiography during the same period were randomly selected. Exclusion criteria included serious lung disease, thyroid dysfunction, acute infection, rheumatism, immune diseases, and chronic inflammatory disease. In this study, all enrolled patients underwent clinical evaluation and laboratory screening, and informed consent was obtained from all patients. Past medical history and clinical data, including age, gender, hypertension, diabetes, creatinine level, and cardiac function were all collected. The condition and severity of coronary artery lesions were evaluated by coronary angiography. The stenosis degree of all lesions was completed through at least two sections, and the Gensini score was calculated. The arrhythmia was mainly evaluated using 12-lead ECG and Holter monitor. SPSS 26 software was used for data analysis. Using Student's *t*-test numerical variables were compared between the study groups for independent samples. Chi square (*χ*^2^) test was carried out for comparing categorical data. *P* values less than 0.05 were regarded as statistically significant.

## 3. Results

A sum of 79 patients enrolled in the study comprising the severe coronary artery lesion group of 39 people and severe coronary lesions combined with new-onset tumor group of 40 people. There was no statistical significance between the two groups in relation to age, gender, hypertension, diabetes, LVEF, Gensini scores, and so on (*P* > 0.05) which is shown in Table 1. We observed in our study that the incidence of arrhythmia in the severe coronary artery disease group with new-onset tumor was 60% and another group was 25.6%. This difference between the two groups was statistically significant (*P* < 0.05), which is shown in Table 2. Attention should be paid to arrhythmia before the tumor is treated.

## 4. Discussion

In our study of severe coronary artery disease patients with new-onset tumor, the final results show a higher incidence of cardiac arrhythmia than the patients without tumor complication. Although the association of cardiac arrhythmia with coronary artery disease (CAD) or cancer has long been paid attention to, there still exists little research regarding the relation between the severity of CAD and the prevalence of cardiac arrhythmia in new-onset cancer patients. The etiology of arrhythmias in cancer patients has not been clear yet, despite the anticancer therapy factor, which may be a result of the direct cardiac involvement of the primary cancer or metastasis to heart [8, 9]. Cancer itself is able to lead to arrhythmia, especially atrial fibrillation/flutter (AF/AFL) and ventricular arrhythmia. There is some hypothesis to explain the mechanisms of arrhythmias in cancer patients on the cellular level, including abnormal calcium homeostasis, mitochondrial injury, and cardiac apoptosis [10–12].

In this study, as previously mentioned, arrhythmia, especially atrial fibrillation and ventricular premature beats, was linked to a new-onset cancer group with a higher number of diseased coronary vessels. Repetitive arrhythmia occurred in 60 percent of multivessel CAD patients with new-onset cancer compared with 25.6 percent of those without cancer. The incidence of AF and premature beats was also nearly twofold in the former group. AF is regarded as a highly prevalent complication among patients with cancer history [13]. In addition, a previous study shows a remarkably higher prevalence of multivessel disease in patients with atrial fibrillation [9]. This is in accordance with the findings of ours. This observation is consistent with previous studies, which explored the extent of CAD in patients with AF [14–16]. Since patients with severe coronary artery disease are prone to have poor left ventricular pump function, cancer may somehow accelerate the process of heart failure. [14, 17]. This susceptibility may be related to gathering of cardiovascular risk factors in cancer patients and possibly a shared physiopathology that leads to both malignancy and CAD development in new-onset oncological patients who have not experienced the antineoplastic therapy. All of these mentioned cardiac pathologies are known to drive the incidence of AF [17, 18]. So, severe cardiac functional insufficiency seems more likely to be a trigger for atrial fibrillation in this group as well [14].

Our study revealed no significant difference in the incidence of ventricular arrhythmia between two groups. Previous study shows that ventricular fibrillation is related to a larger amount of ischemic myocardium caused by occlusion of major coronary arteries and lower remnant blood flow in myocardial infarction [19, 20]. Furthermore, it has been shown that patients with malignant arrhythmias had greater numbers of diseased coronary arteries than those who demonstrated infrequent or no ectopic activity [21, 22]. When the myocardium is invaded by tumor, irritation of the heart conduction system can lead to ventricular and supraventricular ectopy [8]. However, there were few studies which have drawn attention to the association between cancer-related ventricular arrhythmia and the extent of coronary vascular involvement.

Severe coronary artery disease patients with cancer might have a higher risk for being amalgamated with cardiac arrhythmias, and attributing this finding to mere coincidence appears inconsequent. The molecular mechanism of cancer-induced cardiac electrophysiology disorder has not been clear yet [23]. Moreover, neurohormonal activation might stimulate atrial or ventricular arrhythmias in CAD with cancer because it has already been shown in previous studies [23, 24]. Larger sample sizes are needed to confirm the effect of cancer on the cardiac arrhythmia in severe CAD patients.

## 5. Conclusion

In our study, patients with severe coronary artery disease complicated with new-onset tumor had higher a incidence of arrhythmia than in patients with severe coronary artery disease alone. Tumor may be a health hazard of cardiac arrhythmia in those patients, and morden medicine should pay more attention to it before cancer treatment.

## Figures and Tables

**Table 1:** Baseline demographic characteristics.

Characteristics	Severe coronary artery lesions combined with new-onset tumor (*n* = 40)	Severe coronary artery lesion group (*n* = 39)	*P* value
Age	70.83 ± 9.14	68.90 ± 11.97	0.423
Gender, male (*n*)	33 (82.5%)	28 (71.8%)	0.257
Hypertension (*n*)	28 (70%)	30 (76.99%)	0.486
Diabetes (*n*)	15 (37.5%)	13 (33.3%)	0.699
Smoking (*n*)	17 (42.5%)	15 (38.5%)	0.715
Creatinine (*µ*mol/L)	124.95 ± 162.93	99.38 ± 91.99	0.395
hs-CRP	14.28 ± 28.85	8.57 ± 16.94	0.282
LVEF (%)	62.03 ± 9.15	63.13 ± 10.39	0.618
LA (mm)	36.80 ± 4.60	36.33 ± 6.27	0.707
Gensini score	54.94 ± 26.42	55.78 ± 34.90	0.905

**Table 2:** Comparison of prevalence in arrhythmia between two groups.

	Severe coronary artery lesions combined with new-onset tumor (*n* = 40)	Severe coronary artery lesion group (*n* = 39)	Chi square (*χ*^2^) test
Prevalence of arrhythmia (*n*)	24 (60%)	10 (25.6%)	*P*=0.002

## Data Availability

The data used to support the findings of this study are available from the corresponding author upon request.
